# Constructing lactylation-related genes prognostic model to effectively predict the disease-free survival and treatment responsiveness in prostate cancer based on machine learning

**DOI:** 10.3389/fgene.2024.1343140

**Published:** 2024-03-19

**Authors:** Jinyou Pan, Jianpeng Zhang, Jingwei Lin, Yinxin Cai, Zhigang Zhao

**Affiliations:** Department of Urology, The First Affiliated Hospital of Guangzhou Medical University, Guangdong Provincial Key Laboratory of Urology, Guangdong Engineering Research Center of Urinary Minimally Invasive Surgery Robot and Intelligent Equipment, Guangzhou Institute of Urology, Guangzhou, China

**Keywords:** lactylation, prostate cancer, prognostic, tumor immune environment, machine learning

## Abstract

**Background:** Prostate cancer (PCa) is one of the most common malignancies in men with a poor prognosis. It is therefore of great clinical importance to find reliable prognostic indicators for PCa. Many studies have revealed the pivotal role of protein lactylation in tumor development and progression. This research aims to analyze the effect of lactylation-related genes on PCa prognosis.

**Methods:** By downloading mRNA-Seq data of TCGA PCa, we obtained the differential genes related to lactylation in PCa. Five machine learning algorithms were used to screen for lactylation-related key genes for PCa, then the five overlapping key genes were used to construct a survival prognostic model by lasso cox regression analysis. Furthermore, the relationships between the model and related pathways, tumor mutation and immune cell subpopulations, and drug sensitivity were explored. Moreover, two risk groups were established according to the risk score calculated by the five lactylation-related genes (LRGs). Subsequently, a nomogram scoring system was established to predict disease-free survival (DFS) of patients by combining clinicopathological features and lactylation-related risk scores. In addition, the mRNA expression levels of five genes were verified in PCa cell lines by qPCR.

**Results:** We identified 5 key LRGs (ALDOA, DDX39A, H2AX, KIF2C, RACGAP1) and constructed the LRGs prognostic model. The AUC values for 1 -, 3 -, and 5-year DFS in the TCGA dataset were 0.762, 0.745, and 0.709, respectively. The risk score was found a better predictor of DFS than traditional clinicopathological features in PCa. A nomogram that combined the risk score with clinical variables accurately predicted the outcome of the patients. The PCa patients in the high-risk group have a higher proportion of regulatory T cells and M2 macrophage, a higher tumor mutation burden, and a worse prognosis than those in the low-risk group. The high-risk group had a lower IC50 for certain chemotherapeutic drugs, such as Docetaxel, and Paclitaxel than the low-risk group. Furthermore, five key LRGs were found to be highly expressed in castration-resistant PCa cells.

**Conclusion:** The lactylation-related genes prognostic model can effectively predict the DFS and therapeutic responses in patients with PCa.

## Introduction

Prostate cancer (PCa) is one of the most common malignant tumors of the male genitourinary system. According to the latest cancer clinical data in the United States in 2024, the incidence of prostate cancer in male tumors ranks first, and the mortality rate of prostate cancer ranks second (Siegel et al., 2024). Although the overall survival of prostate cancer is longer than other cancers, the recurrence rate of localized prostate cancer after radical prostatectomy is still high, and the recurrence progresses and eventually causes death. A key challenge in the management of PCa is the clinical heterogeneity that is hard to predict using existing biomarkers (Bahmad et al., 2021). The PCa heterogeneity is caused by genomic and epigenetic changes that have been revealed by examination of prostate tumor tissue samples (Khan et al., 2024). How to identify patients with a high risk of recurrence early, and early intervention treatment may prolong the survival of patients and improve the quality of life of patients. Therefore, developing an effective prognostic signature that is required to improve patient prognosis and guide patients to assess cancer risk, and conduct, precision medicine treatment (such as individualized chemotherapy and immunotherapy), is an urgent issue.

Lactylation is a novel posttranslational modification first reported in 2019, which modulates histones through the addition of lactyl groups to their lysine residues and facilitates specific gene transcription (Zhang et al., 2019). Yang and colleagues observed extensive protein lactylation in HCC tissues by immunostaining and further confirmed that the emulsification mainly occurred on histones and non-histone proteins by mass spectrometry (Yang et al., 2023). Wan et al. reported that protein lactylation widely exists in various human normal tissues and cancer tissues in 2022 Jul (Wan et al., 2022). Protein lactation is a crucial mechanism through which lactate performs its duties and is required in key biological processes such as glycolysis related to cell function (Li et al., 2020), macrophage polarization (Irizarry-Caro et al., 2020), neurodevelopment (Hagihara et al., 2021), and regulation of tumor spread (Yu et al., 2021). Besides, Lactylation promotes DNA damage repair and chemoresistance (Chen et al., 2024). It has been reported that the occurrence and progression of a variety of tumors, such as liver cancer (Gu et al., 2022; Kotsiliti, 2023), breast cancer (Deng and Liao, 2023; Pandkar et al., 2023), colorectal cancer (Li et al., 2023; Zhou et al., 2023), etc., all play an important role. In prostate cancer, histone lactylation affects cancer cell plasticity and leads to transcriptional surges of neuroendocrine genes (He et al., 2023). It has been reported that evodiamine blocked histone lactylation in PCa cells, further enhancing Sema3A transcription while inhibiting that of PD-L1 (Yu et al., 2023). Besides, HIF1α lactylation enhances KIAA1199 transcription to promote angiogenesis and vasculogenic mimicry in PCa (Luo et al., 2022). Based on these inspiring findings, we determined that these lactylation-related genes (LRGs) were closely connected to prostate cancer cell malignancy and spreading. Therefore, systematic evaluation of the relationship between differentially expressed LRGs and the prognosis, immune microenvironment, and treatment response of PCa is still worth further exploration. Here, we collected the lactylation-related data published thus far and systematically analyzed the expression levels of these genes in different databases to develop a novel prognostic signature based on LRGs to systematically explore the relationships between the signature and clinicopathological characteristics and disease progression in patients with PCa. In addition, we further investigated its correlation with the tumor microenvironment (TME), mutation profiles, and the patient’s response to immunotherapy and chemotherapy in PCa. A lactylation-related gene signature that effectively predicts both prognosis and treatment responsiveness in PCa has not been reported to date.

## Materials and methods

### Data acquisition

**FIGURE 1:**
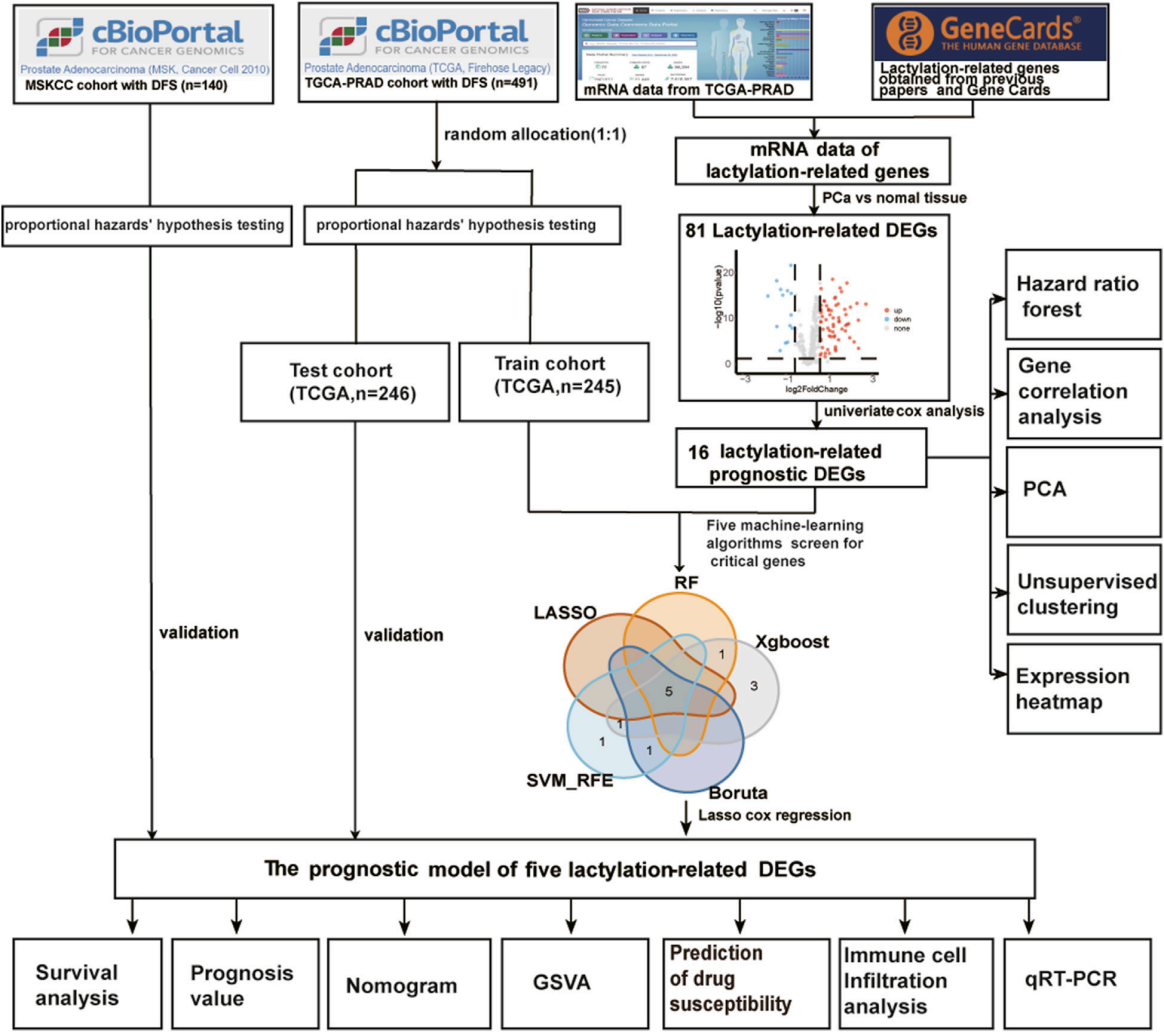
The main workflow of the study. DEGs, differentially expressed genes; DFS, disease-free survival; RF, random forest; LASSO, the least absolute shrinkage and selection operator; XGBoost, the optimal extreme gradient boosting; SVM-RFE, Support Vector Machine Recursive Feature Elimination; PCA, principal component analysis; GSVA, gene set variation analysis.

Figure 1 displays the overall procedure of the study. The mRNA transcriptome profiles of 502 prostate cancer samples and 52 normal samples were downloaded from The Cancer Genome Atlas (TCGA) database, https://protal.gdc.cancer.gov/(accessed on 7 May 2023). The corresponding clinical information for TCGA-PRAD was download from Prostate Adenocarcinoma (TCGA, Firehose Legacy) and could be directly downloaded from the following: http://www.cbioportal.org/. When a patient would correspond to more than one sample, only samples with a sample sequence number (Vial) of A were retained, and samples with no disease-free survival were removed, resulting in 491 tumor samples. External validation data were downloaded from Prostate Adenocarcinoma (MSK, Cancer Cell 2010), and 140 prostate cancer samples remained after prostate Adenocarcinoma samples without disease-free survival were deleted. A scale method-based normalization was performed with the gene expression profiles using the R package “limma” (v4.2.2). The lactylation-related genes were gathered from Gene Card (lactylation was entered into the Gene Card website to search for 92 genes, and 79 related genes with Relevance score >0.6 were obtained) and from previously published studies (Zhang et al., 2019; Moreno-Yruela et al., 2022; Cheng et al., 2023). We got a total gene set of 400 lactylation-related genes (LRGs) after deleting duplicate genes, found in Supplementary Table S1.

### Identification of differentially expressed and prognostic genes

The mRNA levels of lactation-modified genes were extracted from tumor samples and adjacent tissue samples in the TCGA prostate cancer data for difference analysis by R package “limma” (Ritchie et al., 2015). The criteria for differentially expressed genes (DEGs) identification were a false discovery rate (FDR) < 0.05 and |log2 FC| ≥ 0.585. By applying these criteria, genes expressed at more than 1.5-fold levels in tumor tissues and adjacent tissues were screened out at a false discovery rate of less than 0.05. Univariate Cox analysis to assess the association between disease-free survival (DFS) status was performed with a threshold of *p* < 0.05, and 16 prognosis-related genes were screened out. The PCA and t-SNE analysis were applied using the R packages “Rtsne” (v0.16) and “ggplot2” (v3.4.0).

### Unsupervised cluster analysis

Unsupervised cluster analysis was performed with the expression of 16 prognostic genes in them for patients (TCGA, n = 491) using the “ConsensusClusterPlu” R package (Wilkerson and Hayes, 2010). The proportion of items per sample was 0.8, and the proportion of features per sample was 1. Partition Around Medoids was used. The adjacency distance matrix was determined as (Pearson correlation coefficient). Default settings were used for other parameters.

### Selection of key genes and construction of the prognostic model

The 491 prostate cancer patients in the TCGA cohort were randomly divided into training cohort and test cohort at a ratio of 1:1. To check whether the TCGA training set, test cohort, and MSKCC cohort meet the conditions for the Cox proportional hazards model, we performed Schoenfeld residual check on TCGA training cohort, TCGA test cohort, and MSKCC cohort to check whether the proportional hazards’ assumption was met by R package “survminer” and “survival”. Five different machine learning algorithms: the least absolute shrinkage and selection operator (LASSO), random forest, the optimal extreme gradient boosting (XGBoost), Boruta algorithm, and Support Vector Machine Recursive Feature Elimination (SVM-RFE) were used for the eigengene screening. LASSO was implemented as a dimensionality reduction method to perform variable screening and complexity adjustment while fitting a generalized linear model. LASSO analysis was implemented with a penalty parameter utilizing a 10-fold cross-verification via the “glmnet” package. The XGBoost uses a gradient boosting framework and improves on objective optimization function which is to optimize the loss function and complexity punishment by the R package “xgboost”. The Boruta algorithm was used to reduce the dimension of molecules according to clusters and to distinguish important features from irrelevant features (R-package Boruta, +1 to the FPKM matrix value, and then take the log2, finally the function scale was used to standardize the matrix, doTrace = 2, ntree = 500). The SVM-RFE is a supervised machine learning method for support vectors that can find the best variables by halving the features each round when there are many features by the R package “e1071”. LASSO‐penalized Cox regression analysis was performed on prognostic lactylation-related genes to find the potential gene set suitable for the prognostic signature, and the optimal penalty parameter λ and gene coefficients of the risk scoring formula were obtained (Friedman et al., 2010). The risk score model trained from the TCGA data was constructed as follows:

$$\text{Riskscore}=\sum_{i=1}^{n}\left(\exp\times \text{coef}\right)$$

where N is the number of model genes; exp represents the expression value of genes; and coef is the coefficient of each gene. The prognostic scoring system for PCa patients was developed based on a linear combination of regression coefficients derived from the LASSO Cox regression analysis coefficients multiplied by the expression levels of genes, and then patients were divided into high-risk and low-risk groups according to the median risk value. Kaplan-Meier analysis was conducted to evaluate differences in disease-free survival (DFS) time between high-risk and low-risk groups. To evaluate the stability and specificity of the prognostic model, the R package “survivalROC” was employed to perform receiver operating characteristic (ROC) analysis and calculate the value of the area under the curve (AUC) (Heagerty et al., 2000). The TCGA test cohort and MSKCC cohort were used to validate the prognostic model.

### Gene set variation analysis (GSVA)

“c2. Kegg. v7.4. symbols” and “c5. go.v7.4. symbols” gene sets were applied to performed GSVA to investigate the difference of the biological function between high- and low-risk groups in TCGA cohort by the R package “GSVA”.

### Analysis of tumor mutation burden and immune cell infiltration

R package “Maftools” was used to analyze and visualize the somatic variation data (MAF files) of high- and low-risk group prostate cancer samples in TCGA. We utilized CIBERSORT, ESTIMATE, MCP counter, TIMER algorithms, Immunophenoscore algorithms (IPS), EPIC algorithm, and xCell algorithms (Aran et al., 2017; Charoentong et al., 2017; Li et al., 2017; Racle et al., 2017; Chen et al., 2018; Dienstmann et al., 2019; Xiang et al., 2021) to evaluate the immune cell levels of the two groups. Furthermore, we applied the ssGSEA algorithm to quantify the subgroups of the infiltrating immune cells between the two groups. The differences in immune response under different algorithms were revealed using a Heatmap.

### Drug sensitivity prediction of the risk model

We used the R package “OncoPredict” (Maeser et al., 2021) to predict the half-maximal inhibitory concentration (IC50) of 198 drugs based on the Genomics of Drug Sensitivity in Cancer (GDSC) databases *in vivo* drug responses between high- and low-risk groups.

### Construction and evaluation of the nomogram

With the “rms” and “survival” packages in R, a nomogram for predicting the 1-, 3-, and 5-year disease-free survival of PCa was constructed using the risk model with clinicopathological parameters such as T-stage, risk score, and Gleason score. To evaluate the nomogram’s effectiveness, the Calibration curve and time-dependent ROC curve analysis were performed.

### Cell lines and cell culture

Human prostate cancer cells (LNCAP, PC3, C4-2, 22Rv1, DU145) were purchased from the Cell Bank of Type Culture Collection of the Chinese Academy of Sciences, Shanghai Institute of Cell Biology, Chinese Academy of Sciences. All PC cell lines were cultured in RP1640 medium (RP1640, Gibco) supplemented with 10% fetal bovine serum (FBS, Gibco), and streptomycin at 37 °C in 5% CO2.

### RNA extraction and quantitative analysis

Total RNA was extracted from cell lines with TRIzol Reagent (Invitrogen, USA). Total RNA was reverse-transcribed into cDNA with PrimeScript RT Master Mix (Takara, USA) and then used to perform quantitative real-time PCR (qRT–PCR) with SYBR qPCR Master Mix (Vazyme, China). GAPDH was used as an internal control for gene quantification. The 2^−ΔΔCT^ was calculated for every sample and normalized to GAPDH. The primer sequences used are shown in Supplementary Table S2.

### Statistical analysis

All statistical analyses were conducted based on R (v4.2.3). Statistical significance was defined as *p*-value <0.05 when there is no special description for the above methods. Statistical significance is indicated with asterisks (*). A two-sided *p*-value of <0.05 was considered statistically significant (*p < 0.05, **p < 0.01, ***p < 0.001, ****p < 0.0001).

## Results

### Identifying prognostic lactylation-related genes and subtypes in PCa

**FIGURE 2:**
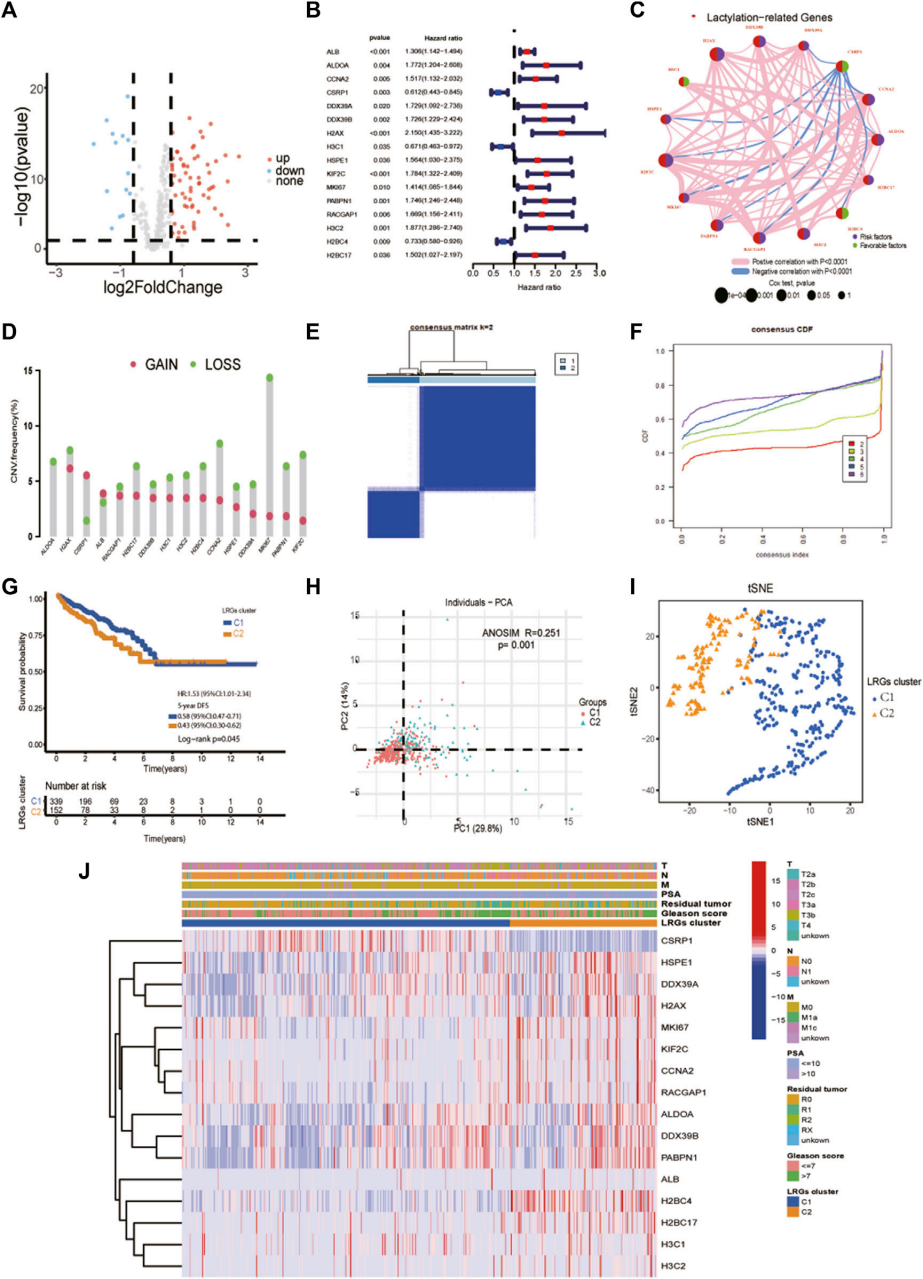
Identifying prognostic lactylation-related genes and subtypes in PCa. **(A)** A volcano plot of differentially expressed lactylation-related genes in the TCGA cohort; **(B)** Forest plots showing the results of the univariate Cox regression analysis of 16 prognostic differentially expressed lactylation-related genes; **(C)** The co-expression network of 16 prognostic differentially expressed lactylation-related genes. **(D)** CNV status analysis showed a frequent alteration in 16 lactylation-related DEGs. **(E–F)** K = 2 was identified as the optimal value for unsupervised clustering. **(G)** The relation of LRGs molecular subtypes to the disease-free survival of PCa patients. **(H)** Principal component analysis of the entire TCGA cohort. **(I)** The distribution was analyzed by t-SNE of the two subtypes in the TCGA cohort. **(J)** The clinicopathological features and differential expression of LRGs in the two molecular subtypes. T, T stage. N, N stage. M, metastasis status. LRGs cluster, lactylation-related genes cluster. PSA, prostate-specific antigen. *p-value <0.05, **p-value <0.01, ***p-value <0.001.

The clinical and pathological characteristics of PCa patients in the TCGA and MSKCC cohorts are listed in Table 1. Using the R package “limma” with an absolute log2-fold change (FC) ≥ 0.585 and an adjusted *p*-value < 0.05 to perform differential expression analysis, 81 lactylation-related genes were differentially expressed between tumor tissues and adjacent nontumorous tissues in the TCGA cohort (Figure 2A). By univariate Cox analysis of the relationship between 81 differentially expressed genes and disease-free survival (*p* < 0.05) of prostate cancer from TCGA, we obtained 16 of the 81 genes were significantly related to the prognosis of PCa (Figure 2B). Close correlation was observed among the 16 lactylation-related DEGs (Figure 2C). CNV status analysis showed a frequent alteration in 16 lactylation-related DEGs (Figure 2D). It was noted that ALDOA only had copy number deletion and most alterations were losses in copy number. Unsupervised consensus clustering analysis of 16 prognostic lactylation-related genes could obtain two clusters with two different lactylation signatures (Figures 2E, F). In this study, consensus clustering based on differential expression of lactylation-related genes was achieved using the R package “Consensus ClusterPlus”. The optimal clustering value was k = 2. Kaplan–Meier method suggested that, compared with the DFS of prostate cancer patients in the C1 and C2 lactylation cluster, PCa patients in the C2 lactylation cluster had significantly shorter DFS time (Figure 2G; *p* = 0.045, HR = 1.53, 95% CI = 1.01–2.34). PCA analysis and t-Distributed Stochastic Neighbor Embedding suggested significant differences between the C1 and C2 lactylation clusters (*p* = 0.001) (Figures 2H, I). The heatmap for the association between 16 lactylation-related DEGs and clinicopathological manifestations was also analyzed (Figure 2J).

**TABLE 1:** Characteristics of sample cohorts used for the analysis of DFS.

Characteristics cohort	TCGA cohort			MSKCC cohort
	TCGA training cohort		TCGA test cohort	
Total number of patients	245		246	140
	pT2	82 (33.5%	95 (38.6%)	86 (61.4%)
T-stage	pT3	139 (56.7%)	144 (58.5%)	47 (33.6%)
	pT4	5 (6.1%)	5 (2%)	7 (5%)
	Not available	19 (13.7%)	2 (0.8%)	0
	pN0	170 (69.4%)	171 (69.5%)	
N-stage	pN1	38 (15.5%)	40 (16.3%)	
	Not available	37 (15.1%)	35 (14.2%)	140 (100%)
	M0	224 (91.4%)	226 (91.9%)	
Metastasis	M1	2 (0.8%)	0 (0%)	
	Not available	19 (7.8%)	20 (8.1%)	140 (100%)
	≤ 7	141 (57.6%)	149 (60.6%)	117 (83.6%)
Gleason score	> 7	104 (47.4%)	97 (39.4%)	21 (15%)
	Not available	0 (0%)	0 (0%)	2 (1.4%)
	R0	160 (65.3%)	152 (61.8%)	
Residual tumor	R1-2	73 (29.8%)	77 (31,3%)	
	Rx	8 (3.3%)	7 (2.8%)	
	Not available	4 (1.6%)	10 (4.1%)	140 (100%)
	≤ 10	225 (91.8%)	207 (84.1%)	
PSA	> 10	5 (2%)	10 (4.1%)	
	Not available	15 (6.1%)	29 (11.8%)	140 (100%)

### Selection of key LRGs by machine learning and construction of a LRGs prognostic model with good performance

**FIGURE 3:**
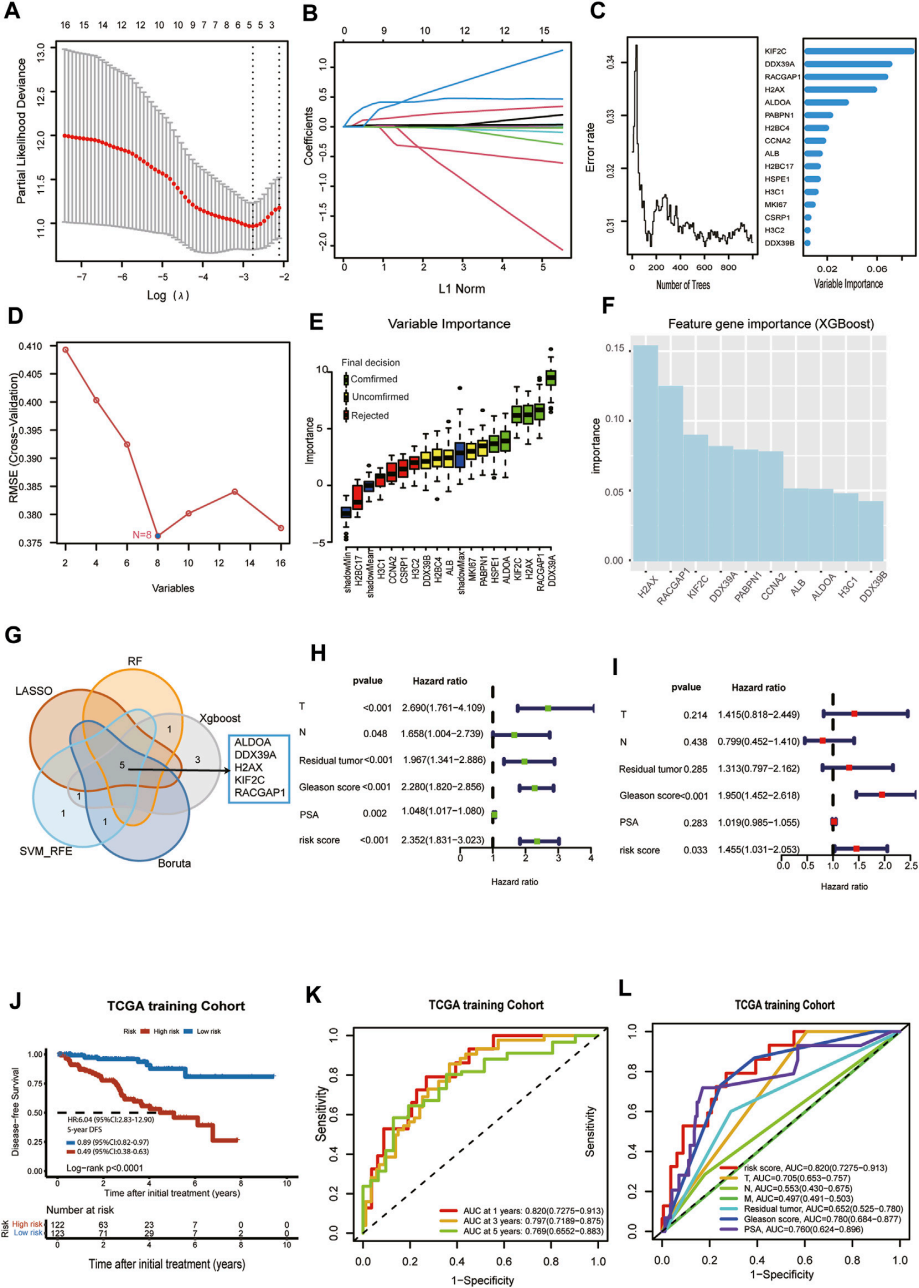
Selection of critical LRGs by machine learning and construction of an LRGs prognostic model with good performance. **(A)**The optimal parameter (lambda) was selected in the LASSO model: dotted vertical lines were drawn at the optimal values using the minimum criteria. **(B)** LASSO coefficient profiles of the candidate lactylation-related genes with nonzero coefficients determined by the optimal lambda. **(C)** The error rate of random survival forest (left panel); out-of-bag variable importance ranking (right panel). **(D)** 8 key genes were identified by the SVM-RFE algorithm. **(E)** 6 key genes were obtained using the Boruta algorithm. **(F)** XGBoost algorithm helped to select 10 key genes. **(G)** Venn diagram. Five overlapping eigengenes were screened out via LASSO regression analysis, random forest, XGBoost, Boruta algorithm, and SVM-RFE. **(H)** Univariate Cox regression in TCGA cohort. **(I)** Multivariate Cox regression in TCGA cohort. **(J)** Kaplan-Meier curve of disease-free survival between patients in high-risk group and low-risk group in TCGA training cohort. **(K)** The 1-, 3- and 5-year AUC of the prognostic signature in TCGA training cohort. **(L)** ROC curves for the risk score and other clinical features in TCGA training cohort.

Prostate cancer patients with DFS in TCGA were randomly divided into a training cohort (n = 245) and test cohort (n = 246) at a ratio of 1:1. Because we need to construct the Cox proportional hazards regression model later, the main premise for constructing the Cox proportional hazards regression model is the assumed hazard ratio. We check each collaborator’s variable (T stage, N stage, metastasis status, PSA, Residual tumor, risk score) is in line with the Cox proportional hazards model assumptions with Schoenfeld residuals. Each schoenfeld individual test p was not statistically significant (*p* > 0.05), and the global schoenfelds were not statistically significant (Supplementary Figure S1). Therefore, we can assume that this Cox model meets the proportional hazards’ assumption. Then, we used LASSO regression analysis, random forest, XGBoost, Boruta algorithm, and SVM-RFE to find key genes associated with DFS in the TCGA training cohort. Five lactylation-related genes, ALDOA, DDX39A, H2AX, KIF2C, and RACGAP1, were identified as key genes by the LASSO regression algorithm. The five variables were obtained based on the optimal value of λ = 0.069 (Figure 3A), and the lowest partial likelihood of deviance is shown in Figure 3B. According to the correlation map between the number of decision trees and the model error, we chose ntree was 600, the error rate of the model tended to stabilize and the importance of variables was ranked according to the VIMP method (Figure 3C). Six key genes, ALDOA, DDX39A, H2AX, KIF2C, RACGAP1, and H2BC4, were selected by the minimum depth variable selection function in the R package “randomForestSRC”. The SVM-RFE algorithm identified 8 key genes significantly associated with the DFS (Figure 3D). The Boruta algorithm identified 6 key genes significantly associated with the DFS (Figure 3E). XGBoost algorithm screened the 10 most important signature genes affecting the disease-free survival of prostate cancer (Figure 3F). The intersection of the RF, LASSO, SVM-RFE, Boruta, and XGBoost results were shown in a Venn diagram in Figure 3G. We identified 5 overlapping key genes, including ALDOA, DDX39A, H2AX, KIF2C, and RACGAP1. The overlapping key genes screened by the five machine learning algorithms were consistent with those screened by the lasso algorithm. We wanted to use the five signature genes to construct a survival prognosis model. Then, the five key genes were used to construct a survival prognostic model by Lasso Cox regression analysis in the TCGA training set, and validated in the TCGA test set and external validation set MSKCC. The risk score model was as follows: Risk score = (0.0880 × expression level of ALDOA) + (0.0108 × expression level of DDX39A) + (0.0199 × expression level of H2AX) + (0.2848 × expression level of KIF2C) + (0.0181× expression level of RACGAP1). Univariate and multivariate Cox regression analyses demonstrated that T stage, Gleason score, and risk score based on the signature of five lactylation-related genes were independent predictors of prognosis in patients with PCa (Figure 3HI). The patients were divided into high-risk or low-risk groups according to the median value of the risk scores in the TCGA training cohort. KM analysis indicated that significantly poorer DFS in the TCGA training cohort was detected among patients in the high-risk group compared to patients in the Low-risk group (log-rank test, *p* < 0.0001, HR = 6.04,95%CI:2.83-12.9,5-yearsDFS:0.49,95%CI:0.38-0.63) (Figure 3J). A receiver operating characteristic (ROC) curve was constructed to estimate the model and assess the reliability of the risk score, and the areas under the curve (AUCs) in the TCGA training cohort at 1 year, 3 years, and 5 years DFS were 0.82, 0.797 and 0.769, suggesting that the risk model can be useful in predicting prognosis (Figure 3K). The ROC curve analysis shows the risk score’s highly sensitive and specific prognostic performance in the TCGA training cohort (Figure 3L). So, the Prognostic model had a better predictive performance in the TCGA training cohort.

### An internal validation set (TCGA test cohort) and an external validation set (MSKCC cohort) were used to verify the prediction performance of the prognostic model

**FIGURE 4:**
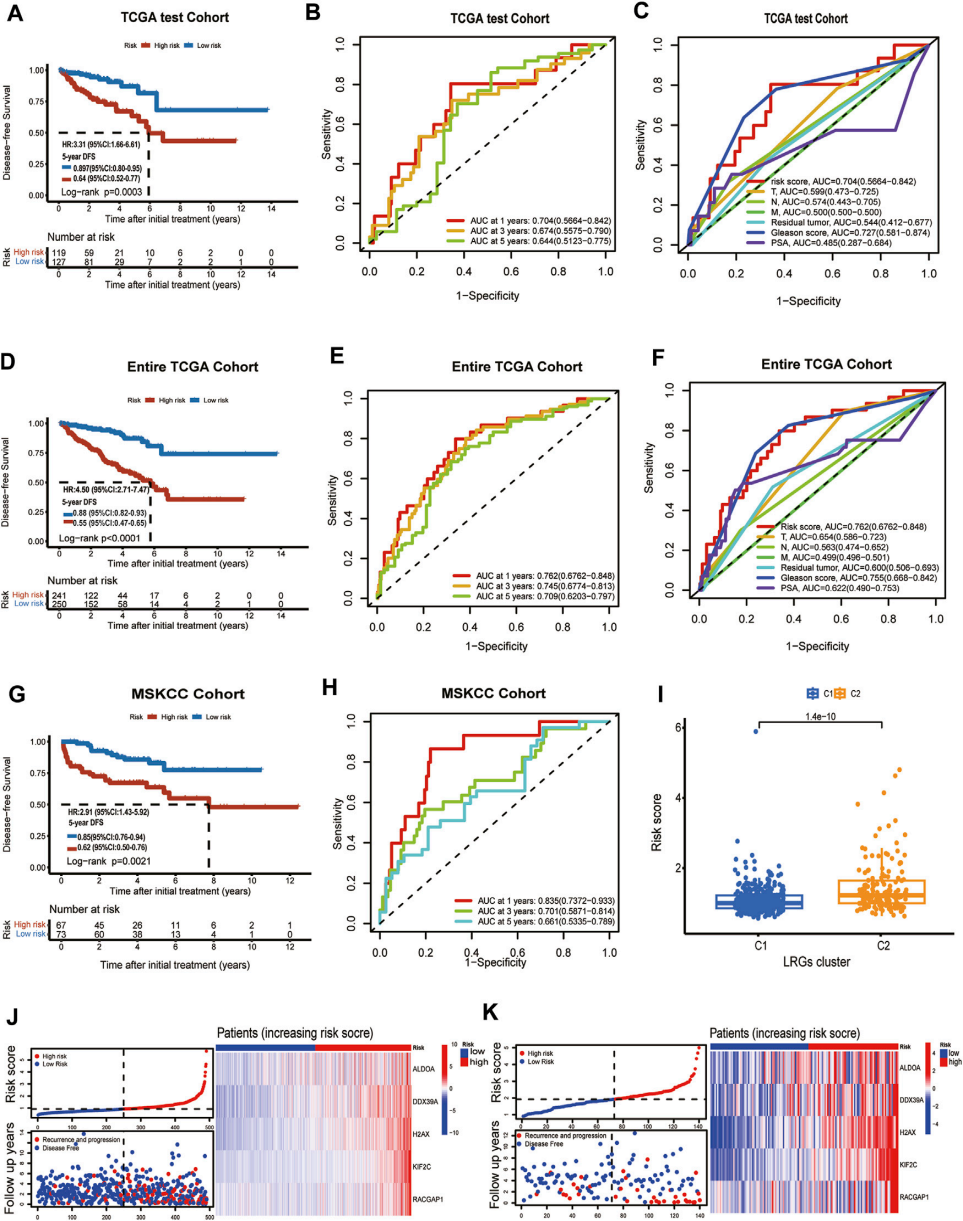
TCGA test cohort and MSKCC cohort were used to validate the prognostic model. **(A)** Kaplan-Meier curve of disease-free survival between patients in high-risk group and low-risk group in TCGA test cohort. **(B)** The 1-, 3- and 5-year AUC of the prognostic model in TCGA test cohort. **(C)** ROC curves for the risk score and other clinical features in TCGA test cohort. **(D)** Kaplan-Meier curve of disease-free survival between patients in high-risk group and low-risk group in entire TCGA cohort. **(E)** The 1-, 3- and 5-year AUC of the prognostic signature in entire TCGA cohort. **(F)** ROC curves for the risk score and other clinical features in entire TCGA cohort. **(G)** Kaplan–Meier analyses demonstrating the prognostic significance of the risk model in MSKCC cohort. **(H)** ROC curves of a prognostic model in MSKCC cohort. **(I)** The box plot depicts the risk score for the different clusters. **(J)** Risk scores distribution, survival status of each patient, and heatmaps of prognostic 5- gene signature in entire TCGA cohort; **(K)** Risk scores distribution, survival status of each patient, and heatmaps of prognostic 5- gene signature in MSKCC cohort.

To verify the prediction model had a powerfully predictive performance in the other cohort, the TCGA test cohort and the MSKCC cohort were used to verify the predictive performance. According to the median value of the risk scores in the TCGA test cohort, the patients were divided into a high-risk group and a low-risk group. KM analysis indicated that significantly poorer DFS in the TCGA test cohort was detected among patients in the high-risk group compared to patients in the Low-risk group (log-rank test, *p* = 0.0003, HR = 3.31,95%CI:1.16-6.61,5-years DFS:0.64,95%CI:0.52-0.77) (Figure 4A). In the TCGA test cohort, the AUC for the 1-year, 3-year, and 5-year DFS were 0.704,0.674 and 0.644 (Figure 4B). The ROC curve analysis shows the risk score’s highly sensitive and specific prognostic performance in the TCGA test cohort (Figure 4C). To better assess its predictive accuracy, the above results were replicated in the entire TCGA cohort. We found that the DFS of the high-risk group was shorter than that of the low-risk group (*p* < 0.001) and the area under the ROC curves (AUC) of the prognostic model for DFS was 0.762 at 1 year, 0.745, at 3 years, and 0.709 at 5 years in entire TCGA cohort (Figures 4D, E). Also, in the ROC curve containing risk score, stage, Gleason score, residual tumor, and prostate-specific antigen (PSA), the AUC value of the risk score was higher than other indicators in the entire TCGA cohort (Figure 4F). Similarly, patients with PCa in MSKCC cohort were classified into a high-risk group and a low-risk groups according to the median risk score. KM analysis indicated that the DFS was shorter in patients with high-risk scores than those with low-risk scores in the MSKCC cohort (log-rank test, *p* = 0.0021, HR = 2.91,95%CI:1.43-5.92,5-years DFS:0.62,95%CI:0.50-0.76) (Figure 4G). The areas under the curve (AUCs) of the ROC curve at 1 year, 3 years, and 5 years DFS were 0.835, 0.701, and 0.661, suggesting good predictive ability (Figure 4H). The risk score was statistically different between TCGA subgroups C1 and C2 (Figure 4I). The risk score of each patient in the entire TCGA cohort and MSKCC cohort was calculated based on the expression levels and regression coefficients of these five genes. The distribution of risk scores and survival status in the entire TCGA cohort and MSKCC cohort is shown in Supplementary Figure S4J; Figure 4K. The risk curves reflect the relationship between the risk score and disease-free survival status of patients with prostate cancer, and we found that the probability of recurrence or progression was higher in the high-risk patients than the low-risk patients.

### Establishment and evaluation of the predictive nomogram

**FIGURE 5:**
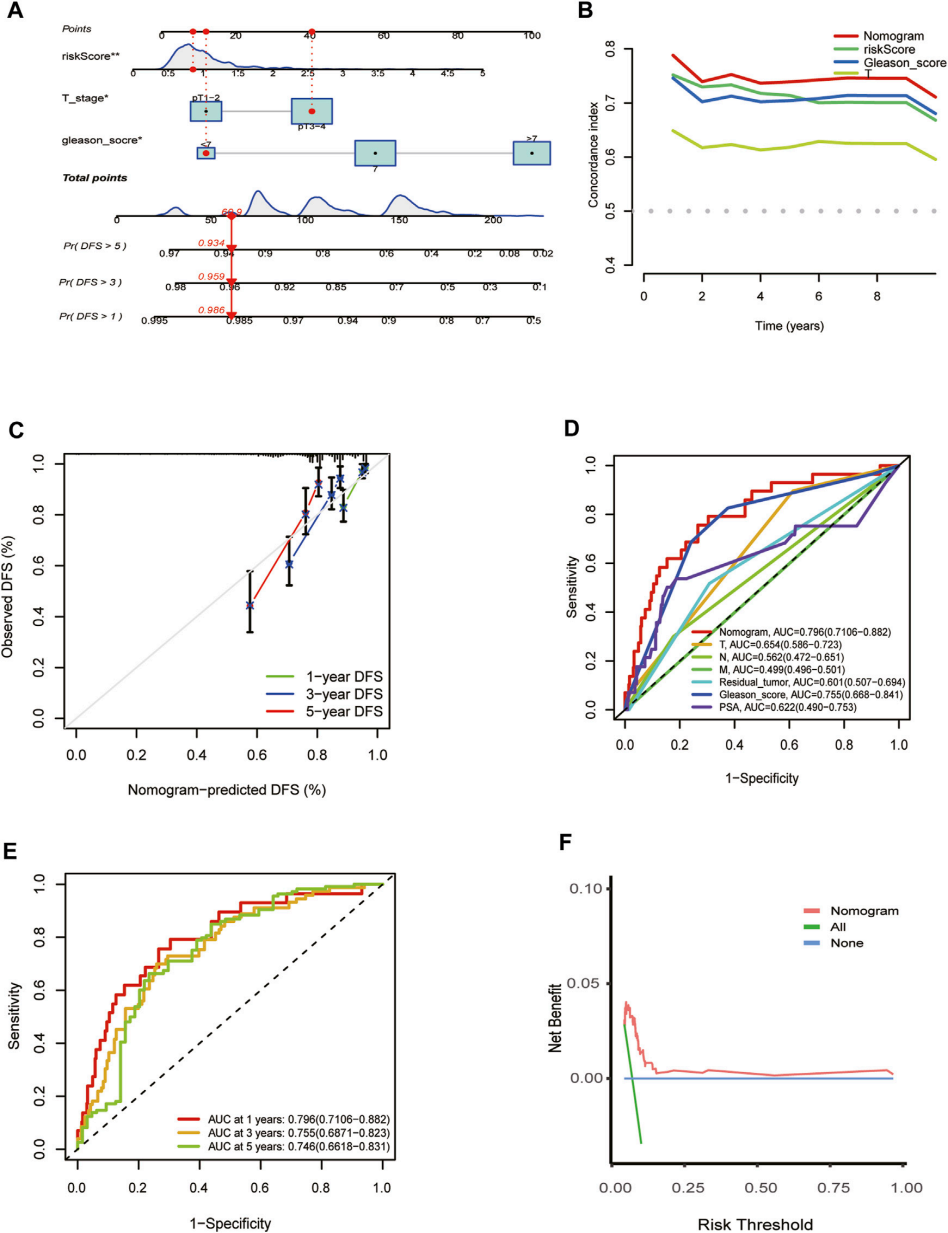
Establishment and evaluation of the predictive nomogram. **(A)** Nomograms for the prognostic prediction of TCGA-PRAD patients. **(B)** C-Index curve analyzed the concordance index of the nomogram, risk score, Gleason score, T. **(C)** 1-year, 3-year, and 5-year DFS calibration chart; **(D)** ROC curves for the nomogram and other clinical features in entire TCGA cohort. **(E)** 1-, 3-, and 5-year DFS area under the ROC curve (AUC) of nomogram in the entire TCGA cohort. **(F)** Decision curve analysis for the nomogram prediction in the TCGA-PRAD cohort.

The independent predictors, including T stage, risk score, and Gleason score, which affect the DFS of PCa patients, were incorporated into the nomogram model (Figure 5A). Time-dependent C-index curves of different variables based on TCGA cohorts show the optimum performance of the nomogram compared with other single factors (Figure 5B). The nomogram AUCs in the TCGA-PRAD cohort for the 1-year, 3-year, and 5-year OS probability were 0.796, 0.755, and 0.746, respectively (Figure 5C). To evaluate the accuracy of this nomogram, the ROC curves were utilized to compare this signature with several available clinical traits. They showed that the predictive value of this nomogram model was more optimal compared to several clinical characteristics. (Figure 5D). Moreover, the calibration curve of the nomogram model showed the actual DFS was close to the predicted DFS (Figure 5E). DCA showed that the nomogram provided a net benefit over different threshold probability ranges, suggesting that it might have potential applications in different contexts (Figure 5F).

### Analysis of tumor immune microenvironment of high- and low-risk groups

**FIGURE 6:**
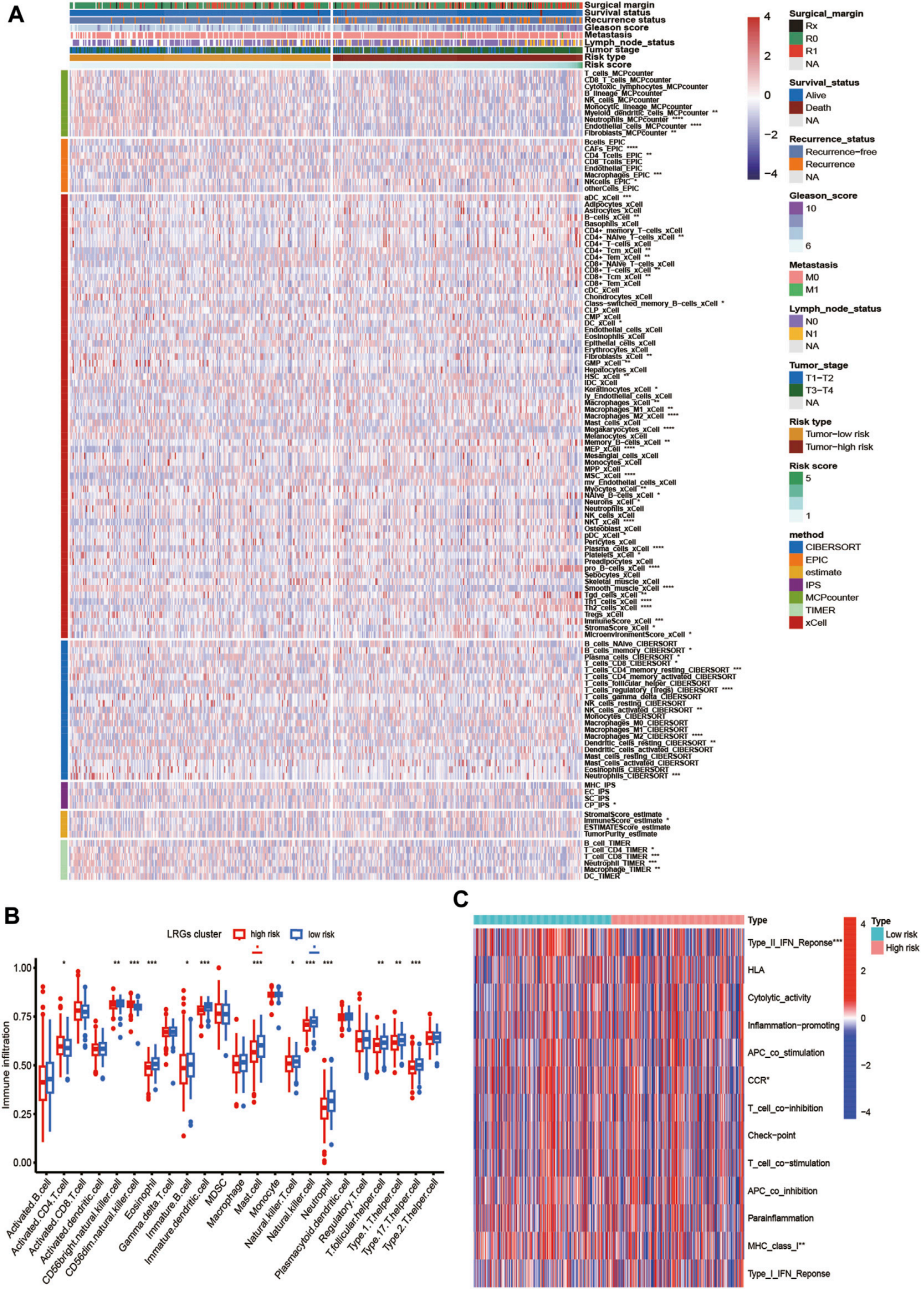
Analysis of tumor immune microenvironment of high- and low-risk groups. **(A)** Heatmap showing the scores of immune and stromal cell infiltrations based on CIBERSORT, ESTIMATE, MCP counter, TIMER algorithms, IPS algorithms, EPIC algorithms and xCell algorithms among high and low-risk group. The statistical difference between the two groups was compared by the Student’s t-test. **(B)** Boxplot of the abundance of immune cells in the high and low-risk groups. The *x*-axis represents the type of immune cells and the *y*-axis represents the level of immune infiltration. Blue represents low-risk group and red represents high-risk group. **(C)** The ssGSEA for the association between immune cell subpopulations and related functions. *p < 0.05, **p < 0.01, ***p < 0.001, ****p < 0.0001, ns, not significant.

**FIGURE 7:**
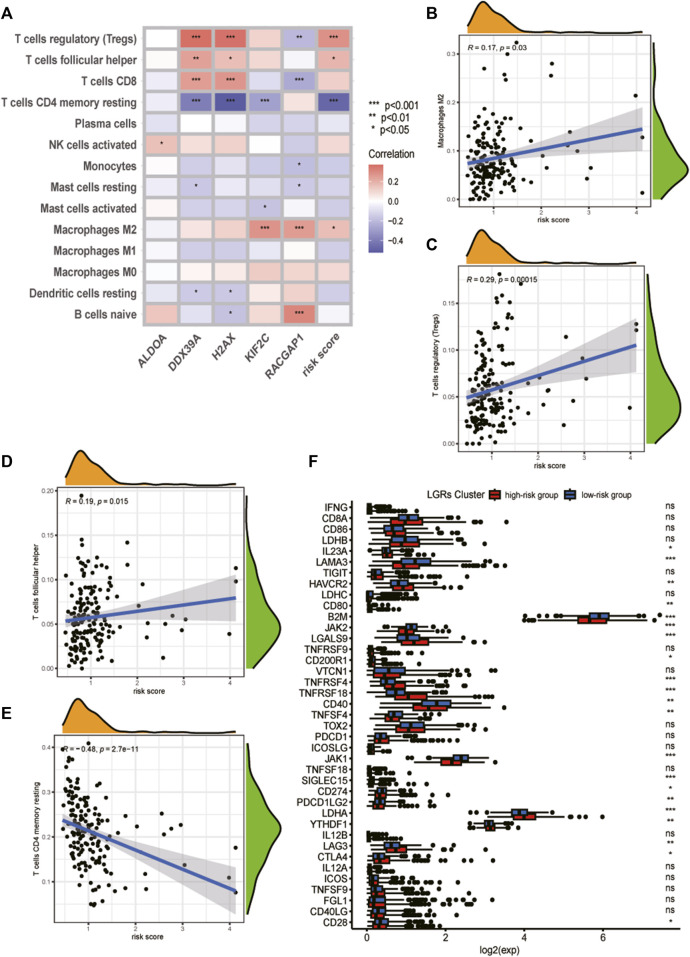
Analysis of tumor immune microenvironment of high- and low-risk groups. **(A)** Heatmap of correlation between the immune cell infiltration and the prognostic genes and risk score. **(B)** Correlation between risk score and M2 Macrophages cells. **(C)** Correlation between risk score and regulatory T cells. **(D)** Correlation between risk score and T cells follicular helper. **(E)** Correlation between risk score and T cells CD4 memory resting. **(F)** Analyses of the expression of immune checkpoints genes in the high and low-risk groups.

First, we estimated the composition of infiltrating immune cells in high- and low-risk groups of the TCGA cohort by CIBERSORT, ESTIMATE, MCP counter, TIMER algorithms, IPS algorithms, EPIC algorithms, and xCell algorithms. The results of Immune cell Infiltration in high and low-risk groups were presented as a heatmap (Figure 6A). From the heatmap, we know that regulatory T cell and M2 Macrophages were significantly higher in the high-risk group compared to the low-risk group. At the same time, Neutrophils and Fibroblasts were substantially lower in the high-risk group. The increase of regulatory T cells and M2 macrophages is conducive to tumor immune escape and tumor recurrence, which suggests that the infiltration of these immune cell subtypes into the tumor microenvironment might significantly impact the prognosis of PCa patients. Based on risk grouping, in terms of immune cells, we discovered that the content of natural killer T cells, natural killer cells, immature dendritic, and neutrophil was significantly lower in patients in the high-risk group compared to the patients in the low-risk group (Figure 6B). Regarding immune functions, the Immune functions of Type II IFN response, CCR, and MHC class I level were significantly downregulated in the patients in the high-risk group compared to those in the low-risk group with ssGSVA algorithm analysis (Figure 6C). Then, we explored the correlation between infiltrating immune cells and risk group and prognostic genes with the CIBERSORT algorithm. We found that the risk score of LRGs was significantly correlated with regulatory T cells, M2 Macrophages, T cells CD4 memory resting, and T cells follicular helper (Figure 7A). Moreover, the risk score had a significantly positive correlation with the regulatory T cells, M2 macrophages, and follicular helper T cells, and it had a significantly negative correlation with the T cells CD4 memory resting (Figures 7B–E). Considering the importance of checkpoint inhibitors in clinical treatment, we further investigated potential changes in immune checkpoint expression between the two groups. Combined with the bar chart showing immune checkpoint expression, where most canonical markers of exhausted T cells, including TIM3/HAVCR2, LAG3 and CTLA4 are highly expressed in the high-risk group of patients. We could conclude that the abundance of exhausted T-cell infiltration in the high-risk group of patients may be higher than that in the low-risk group (Wherry and Kurachi, 2015). Besides, we found that the expression of B2M, JAK1, JAK2, CD40, TNFSF4, and CD28 in the high-risk group was significantly lower than those in the low-risk group (Figure 7F).

### Functional enrichment analysis and drug sensitivity

**FIGURE 8:**
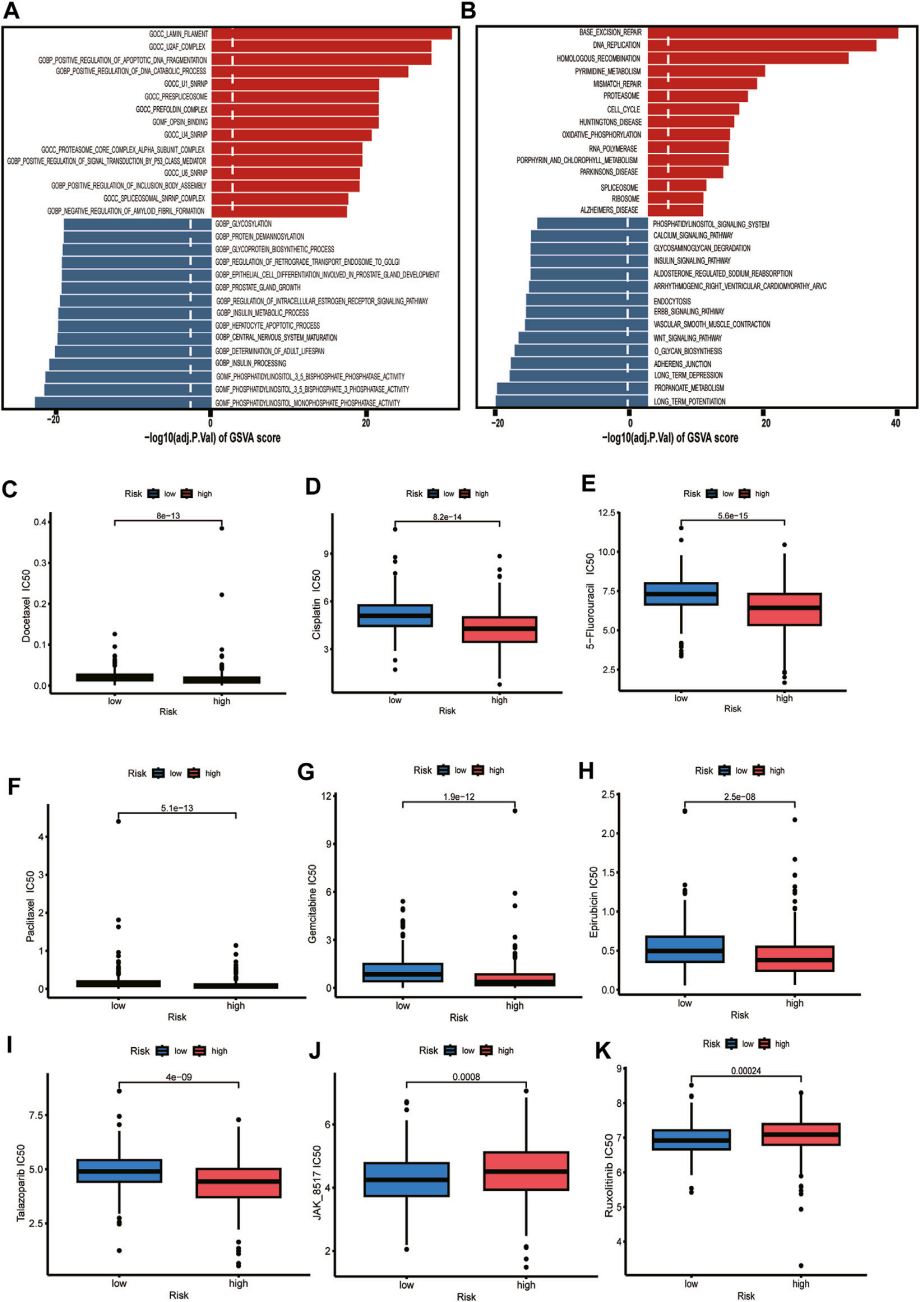
Functional enrichment analysis and drug sensitivity. **(A)** Gene set variation analysis based on the gene ontology biological processes between high-risk and low-risk groups. Dotted line indicates *p*-value less than 0.05. **(B)** Gene set variation analysis based on the KEGG terms between high-risk and low-risk groups. Dotted line indicates *p*-value less than 0.05. **(C–K)** The box plot depicts the drug sensitivity for the different risk groups.

Exploring the functional annotation between high-risk and low-risk subtypes, we found that the high-risk group was significantly enriched in the lamin filament, U2AF complex, positive regulation of apoptotic DNA fragmentation, and positive regulation of DNA catabolic process in the GSVA of gene ontology biological processes (GOBPs) (Figure 8A). KEGG pathway analyses were performed to explore the biological processes and pathways using the gene set variation analysis (GSVA). We found that the high-risk group was enriched in the base excision repair, DNA replication, homologous recombination, pyrimidine metabolism, mismatch repair, and cell cycle (Figure 8B). According to the above functional enrichment analysis, the risk score was closely related to the DNA synthesis, repairing, degradation, and cell cycle progression of PCa. We further analyzed the response of PCa patients in the TCGA cohort to DNA synthesis and repairing and cell cycle-related chemotherapy drugs (5-Fluorouracil, Cisplatin, Docetaxel, Paclitaxel, Gemcitabine, Epirubicin, Talazoparib). The results revealed that these drugs had lower half maximal (50%) inhibitory concentration (IC50) in patients of the high-risk group, implying that these patients may be more sensitive to these drugs (Figures 8C–I). Based on the immune checkpoint analysis, we know that JAK1 and JAK2 are lower in the high-risk group, which is consistent with our results from the drug prediction analysis that we know that the high-risk group has a worse response to JAK inhibitors (JAK_8517, Ruxolitinib) (Figures 8J, K).

### Relationship between the LRGs signature and clinicopathological traits and mutation landscape

**FIGURE 9:**
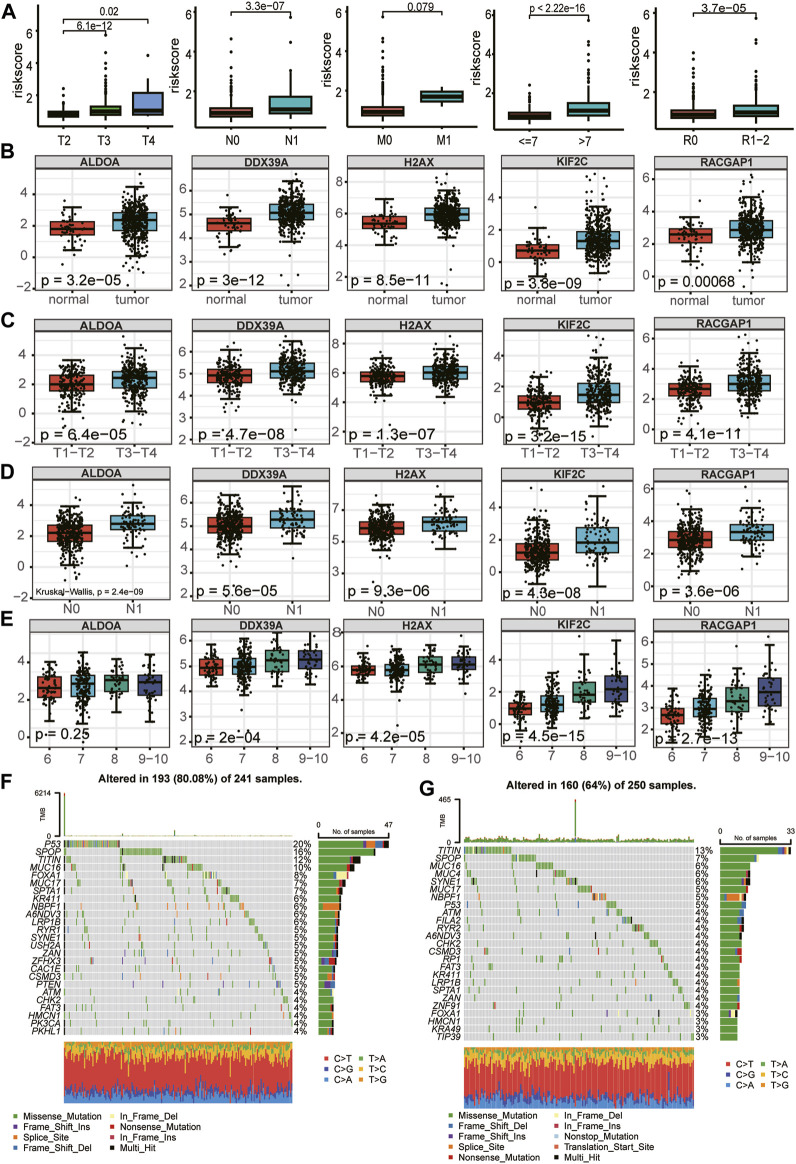
Relationship between the five LRGs and clinicopathological traits and mutation landscape. **(A)** The abundance of the risk score calculated by LRG signature in different clinicopathological characteristics, including tumor stage, N stage, and metastasis status, Gleason score, Residual tumor. **(B–E)** Relationships between each gene of the LRG signature and clinicopathological characteristics of TCGA-PRAD patients, including tumor stage, N stage, and Gleason score. The Wilcoxon test was used for double terms, and the Kruskal test was used for multiple terms. **(F)** SNV waterfall plot of TOP25 (mutation frequency) genes in the high-risk group; **(G)**. SNV waterfall plot of TOP25 (mutation frequency) genes in the low-risk group.

First, we evaluated the association between clinicopathological parameters and the risk score further to investigate the clinical relevance of the prognostic model. The risk score increased significantly with increasing Gleason score, T-stage, and N-stage, but there was no statistical difference between the no metastasis (M0) and metastasis (M1) (Figure 9A). In assessing the relationship between expression profiles of the signature genes in PCa tissues and related clinicopathological characteristics based on the TCGA cohort, we further found the 5 essential genes were differentially expressed between tumor tissues and normal tissues and were highly expressed in tumor tissues (Figure 9B). There were differences in the expression of 5 critical genes in different T stages and N stages, and the expression in advanced T stages (T3 + T4) and N1 was higher than that in early T stages (T1+T2) and N0 (Figures 9C, D). Among the five essential genes, except ALDOA, the expression differed in different Gleason scores and increased with the increase in Gleason score (Figure 9E). We investigated the mutation profiles separately in the high- and low-risk groups of PCa patients from the TCGA cohort. Results indicated that Tumor Mutational Burden (TMB) was more widely distributed in the high-risk group, and miscellaneous mutation was the most common variant classification in PCa. The top five most frequent mutation genes in the high-risk group were TP53 (20%), SPOP (16%), TITIN (12%), MUC16(10%), FOXA1 (8%), while genes such as TITIN (13%), SPOP (7%), MUC16 (6%), MUC4(6%), SYNE1 (6%) had the top five mutation frequencies in low-risk group (Figures 9F, G). Previous studies have shown that mutations in these genes are often associated with a poor prognosis for PCa (Shi et al., 2019; Nyquist et al., 2020; Jiang et al., 2021; Zhang et al., 2022).

### The mRNA expression levels of lactylation‐related genes in PCa cells

**FIGURE 10:**
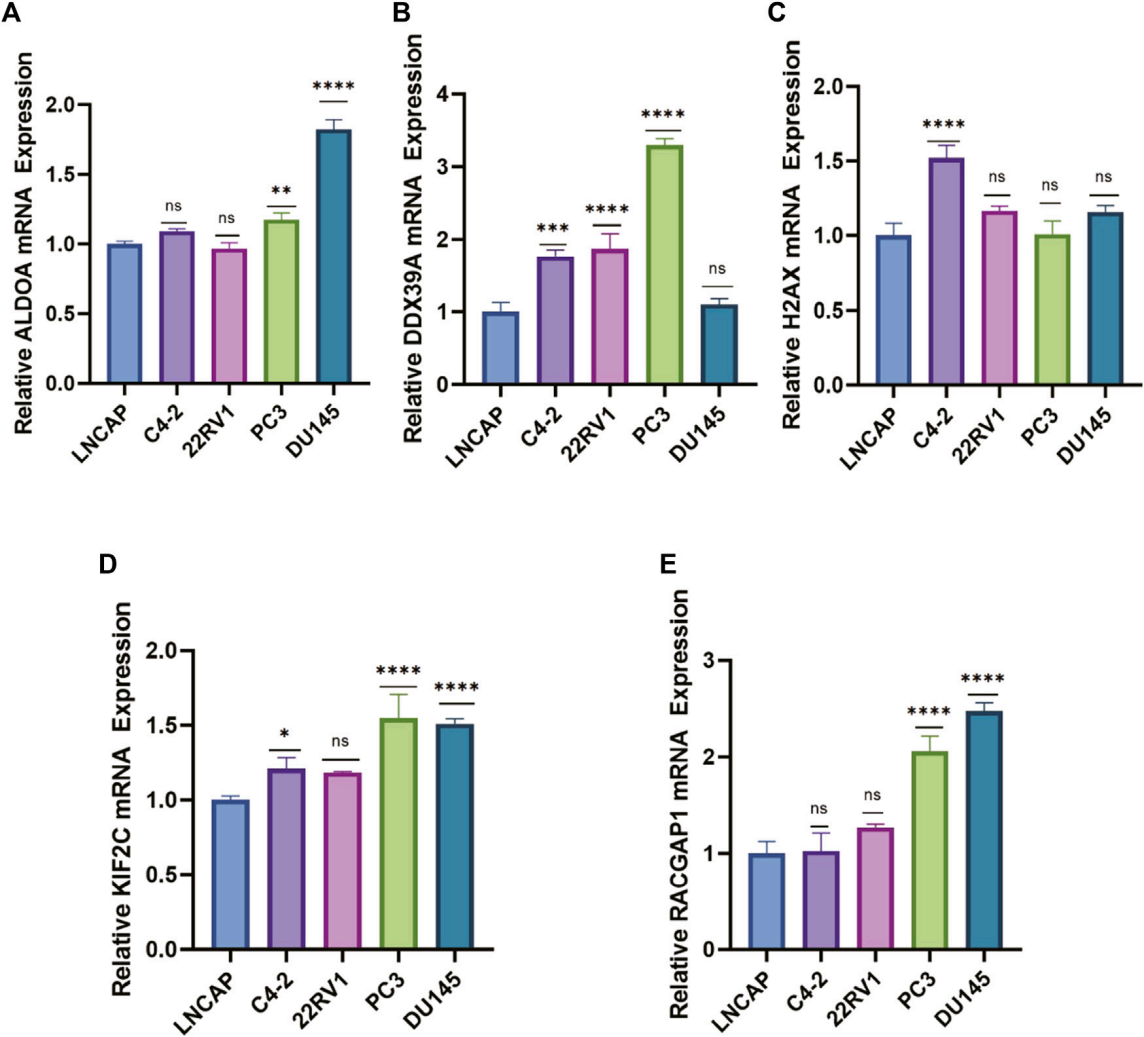
The mRNA expression levels of lactylation‐related genes in PCa cells. **(A–E)** RT–qPCR analysis for the five eigengenes in PCa cell lines. (ns: not significant, *: *p* < 0.05, **: *p* < 0.01, ***: *p* < 0.001, ****: *p* < 0.0001).

To further evaluate the mRNA expression levels of these five genes, we utilized their expression in the cell lines using RT-qPCR. The results indicated that most signature genes were expressed at a relatively high level in castration-resistant prostate cancer cell lines (C4-2, PC3, 22Rv1, DU145) compared with non-castration-resistant prostate cancer cell line (LNCAP) (Figures 10A–E). Castration-resistant prostate cancer cell lines are more malignant than non-castration-resistant prostate cancer cell line. In sum, the high expression of these five key genes is positively correlated with the malignant degree of prostate cancer, which validated the accuracy of our previous bioinformatics analysis.

## Discussion

Prostate cancer is an extremely common malignancy in men. The rates of PCa recurrence and metastasis remain high after radical prostatectomy and were associated with a poor prognosis. Despite the widely used classification system by TNM staging and Gleason score, the heterogeneous nature of PCa poses difficulty to prognosis and therapeutic decision. Therefore, exploring new methods of PCa subtyping has become the most urgent task in bringing precision medicine into reality. Recently, new biomarkers have emerged for the prognosis of cancer patients, but this is the first time to discuss the establishment of a prognostic model related to protein lactation in prostate cancer patients.

Lactylation modification widely occurred on histones and non-histone proteins. Protein lactylation has been found to be related to cell metabolism and cancer immune regulation (Chen et al., 2022). More and more evidence suggests that protein lactylation plays an indispensable role in the development and progression of cancer. In this study, the expression profiles of lactylation-related genes were mined from TCGA PRAD RNA-seq data and were initially analyzed followed by filtering out via differential analysis. Then, 81 LRDEs were subsequent to univariate Cox regression analysis, and a total of 16 prognostic LRGs were identified. The five key LRGs were selected from the 16 prognostic genes by five machine learning algorithms (LASSO regression analysis, random forest, XGBoost, Boruta algorithm, and SVM-RFE). The five key genes (ALDOA, DDX39A, H2AX, KIF2C, RACGAP1) were used to construct a survival prognosis model. Further, the five key LRGs were used to construct a survival prognostic model by Lasso-Cox regression analysis. The predictive performance of the model was verified using the KM curve and ROC curve and the results indicated that the model had good predictive performance. Meanwhile, the nomogram, which was constructed by combining risk score, T stage, and Gleason score, had good accuracy in predicting 1,3,5 years disease-free survival and can be used as a practical and reliable prognostic tool for PCa patients. Among the five essential genes, all were significantly over-expressed in prostate cancer tissues than tumor-adjacent tissue, and their expression was positively correlated with the TNM stage. Many studies reported that these genes played a significant role in the pathogenesis of cancer. ALDOA, a glycolytic enzyme that catalyzes the reversible conversion of fructose-1,6-bisphosphate to glyceraldehyde 3-phosphate and dihydroxyacetone phosphate, was reported to promote the glycolysis of cancer cells and may be significantly associated with the development, metastatic potential, and poor prognosis of various kinds of tumors (Du et al., 2014; Long et al., 2014; Shi et al., 2015; Chang et al., 2017). Nakata et al. reported that DDX39A regulates androgen receptor splice variant AR-V7 generation (Nakata et al., 2017). H2AX coordinates DNA repair following genotoxic stress and double strand breaks (Bonner et al., 2008). The loss of H2AX increased chromosomal instability seen in acute myeloid leukemia, acute lymphoid leukemia, and Head and neck squamous cell may contribute to tumor development, progression, and resistance to therapy in this cancer subtype (Thirman et al., 1993; Parikh et al., 2007). Besides, H2AX promotes metastatic progression in breast cancer cells by preserving glycolysis via hexokinase-2 (Liu et al., 2022). Many studies reported that KIF2C enhanced hepatocellular cancer through the Ras/MAPK and PI3K/Akt signaling pathways and accelerated the growth of cervical cancer by blocking the stimulation of the p53 signaling pathway and activated mTORC1 pathway and promoting tumor cell motility and invasion (An et al., 2021; Moon et al., 2021; Yang et al., 2022). KIFC2 promotes prostate cancer progression by regulating p65 (Liu et al., 2023). A previous study indicated that RACGAP1 regulated the downstream factors of the PI3K/AKT signaling pathway and that the compensatory activation of the PI3K/AKT signaling pathway was closely associated with ADT drug resistance and neuroendocrine differentiation in PCa (Hazar-Rethinam et al., 2015; Song et al., 2023). Besides, the results of qPCR verified that the most signature genes were transcribed at higher levels in more malignant castration-resistant prostate cancer cell lines.

Recently, immune-based treatment has emerged for patients with PCa, which has revolutionized cancer therapy and improved the survival of patients with many types of solid tumors. However, prostate cancer is recognized as a poorly immunogenic tissue with immunological ignorance showing low levels of antigen-presenting process and cytotoxic T-cell activation, high levels of immune checkpoint molecules and immunosuppressive cytokines/chemokines, and recruitment of immunosuppressive cells (Sun, 2021). Due to the immunosuppressive microenvironment and heterogeneous nature of PCa, it is important to identify the molecular subtypes and characterize the TME that can predict response to immunotherapy and identify high-risk patients for early intervention. Therefore, we investigated the status of TME between the high-risk and the low-risk subtypes. With 7 immune algorithms, we found that the proportion of regulatory T cells and M2 macrophages were significantly increased in the high-risk than low-risk group, while Myeloid dendritic cells were significantly decreased in the high-risk group. Previous studies have shown that lactylation modification can regulate inflammatory to reparatory macrophage transition and promote immunosuppression of tumor-infiltrating myeloid cells (Irizarry-Caro et al., 2020; Xiong et al., 2022). M2-polarized macrophages are contributors to many pro-tumorigenic outcomes in cancer through angiogenic and lymphangiogenic regulation, immune suppression, hypoxia induction, tumor cell proliferation, and metastasis (Boutilier and Elsawa, 2021). Sadasivan et al. found that patients with high levels of infiltrating M2 macrophages had an almost 5-fold increased risk of PCa recurrence (Sadasivan et al., 2020). Our results obtained by the immune algorithm were consistent with previous reports that regulatory T cells and M2 macrophages were high in prostate cancer in the high-risk group, so the prognosis of the high-risk group was worse, which may be related to its immunosuppression. Besides, patients in the high-risk group had significantly lower levels of type II IFN response, CCR, and MHC class I compared to patients in the low-risk group by ssGSVA algorithm analysis. These findings strongly implied the potential roles of lactylation -related genes in reshaping the TME in PCa. The results of the GSEA analysis showed that the high-risk score group enriched in positive regulation of DNA catabolic process in gene ontology biological processes (GOBPs), and in base excision repair and DNA replication in KEGG. Then, we explored how patients responded to diverse treatments and tried to offer different recommendations based on LRGs risk groups. Using the R package “OncoPredict” to predict the IC50 of 198 drugs, we found that high-risk group patients were sensitive to DNA synthesis and repairing and cell cycle-related chemotherapy drugs, such as Docetaxel, Cisplatin, 5-Fluorouracil.

Our study systematically evaluated the expression of RLGs and their potential prognostic value in PCa. Moreover, a risk model of five RLGs was established in the TCGA train cohort and validated using the TCGA test cohort and MSKCC cohort. We found that the high-risk group was correlated with elevated TMB and chemotherapy response, and shorter DFS. Undeniably, there are still some limitations of our study. First, since research in the protein lactylation field is still in its preliminary stages, it has not yet fully elucidated how lactylation modification affects protein function in tumor cells. Second, the current analysis is based on RNA level data, and lacking protein level data makes the analysis potentially inaccurate. Due to the lack of appropriate lactylation antibodies, the experimental validation of lactylation proteins is currently hampered by practical problems. Third, the majority of the data are obtained from bioinformatics analysis of the public data.

## Conclusion

We developed a novel lactylation-related prognostic model to predict the prognosis of prostate cancer patients, which had good efficacy in predicting the DFS of PCa patients and provided new insights into personalized therapies for PCa patients. Moreover, further research on these hub genes may contribute to molecular targeted therapy of prostate cancer.

## Data Availability

The original contributions presented in the study are included in the article/Supplementary Material, further inquiries can be directed to the corresponding authors.

## Abbreviations

DEGs, differentially expressed genes; DFS, disease-free survival; LRGs, lactylation-related genes; AUC, area under the curve; ROC, receiver operating characteristic; TME, tumor microenvironment; PCa, prostate cancer; ssGSVA, single sample gene set variation analysis; CCR, chemotactic cytokines receptors; RF, random forest; LASSO, the least absolute shrinkage and selection operator; XGBoost, the optimal extreme gradient boosting; SVM-RFE, Support Vector Machine Recursive Feature Elimination.
