# Supplementary figures and images for: Constructing lactylation-related genes prognostic model to effectively predict the disease-free survival and treatment responsiveness in prostate cancer based on machine learning
Source: Front Genet. 2024 Mar 19;15:1343140. doi: 10.3389/fgene.2024.1343140 (PMC10985269; doi:10.3389/fgene.2024.1343140)

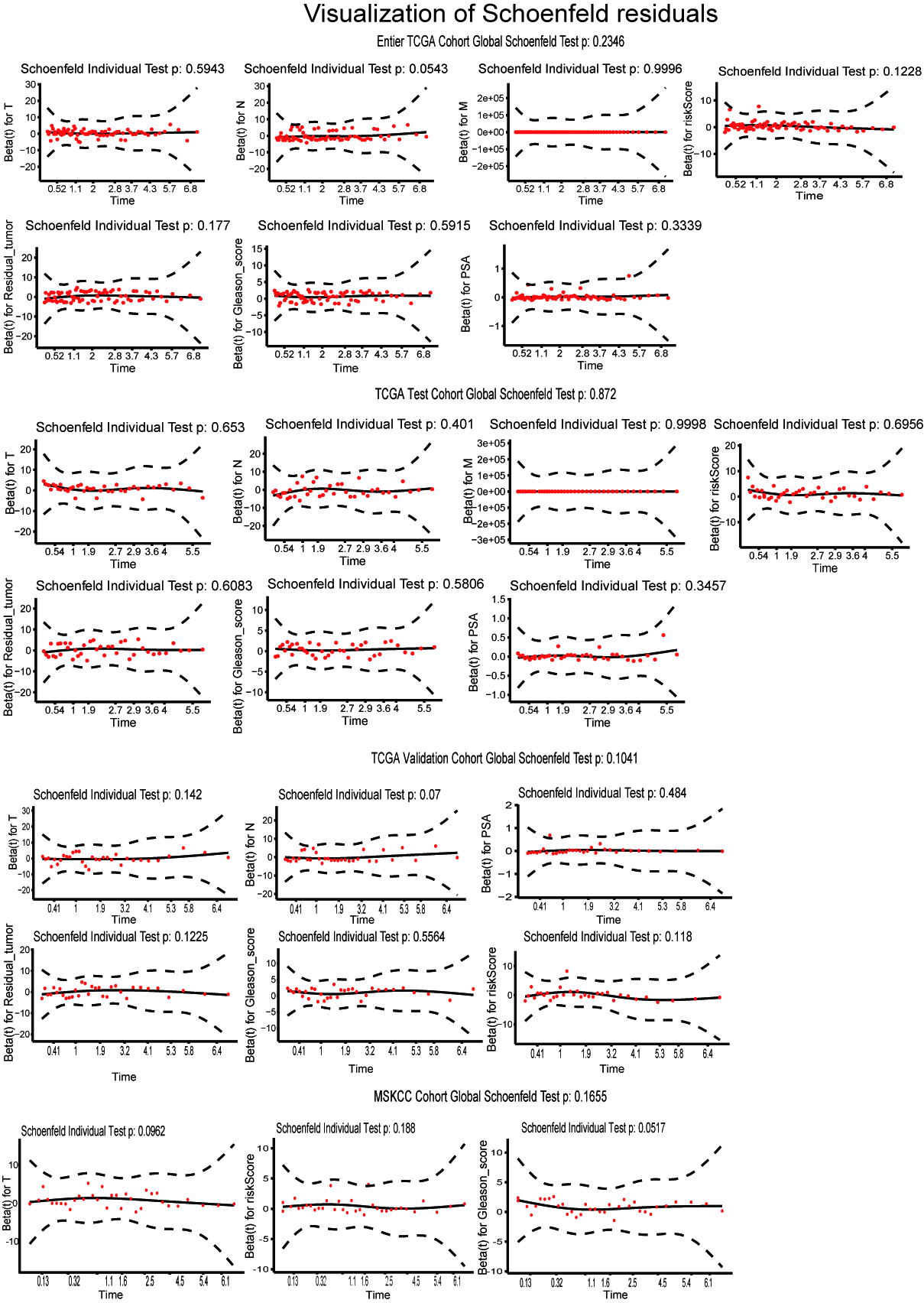

Supplement: Supplementary file 2 [file Image11.tif]
