# Supplementary material for: Constructing lactylation-related genes prognostic model to effectively predict the disease-free survival and treatment responsiveness in prostate cancer based on machine learning
Source: Front Genet. 2024 Mar 19;15:1343140. doi: 10.3389/fgene.2024.1343140 (PMC10985269; doi:10.3389/fgene.2024.1343140)
Supplement: Supplementary file 4 [file Image1.pdf]

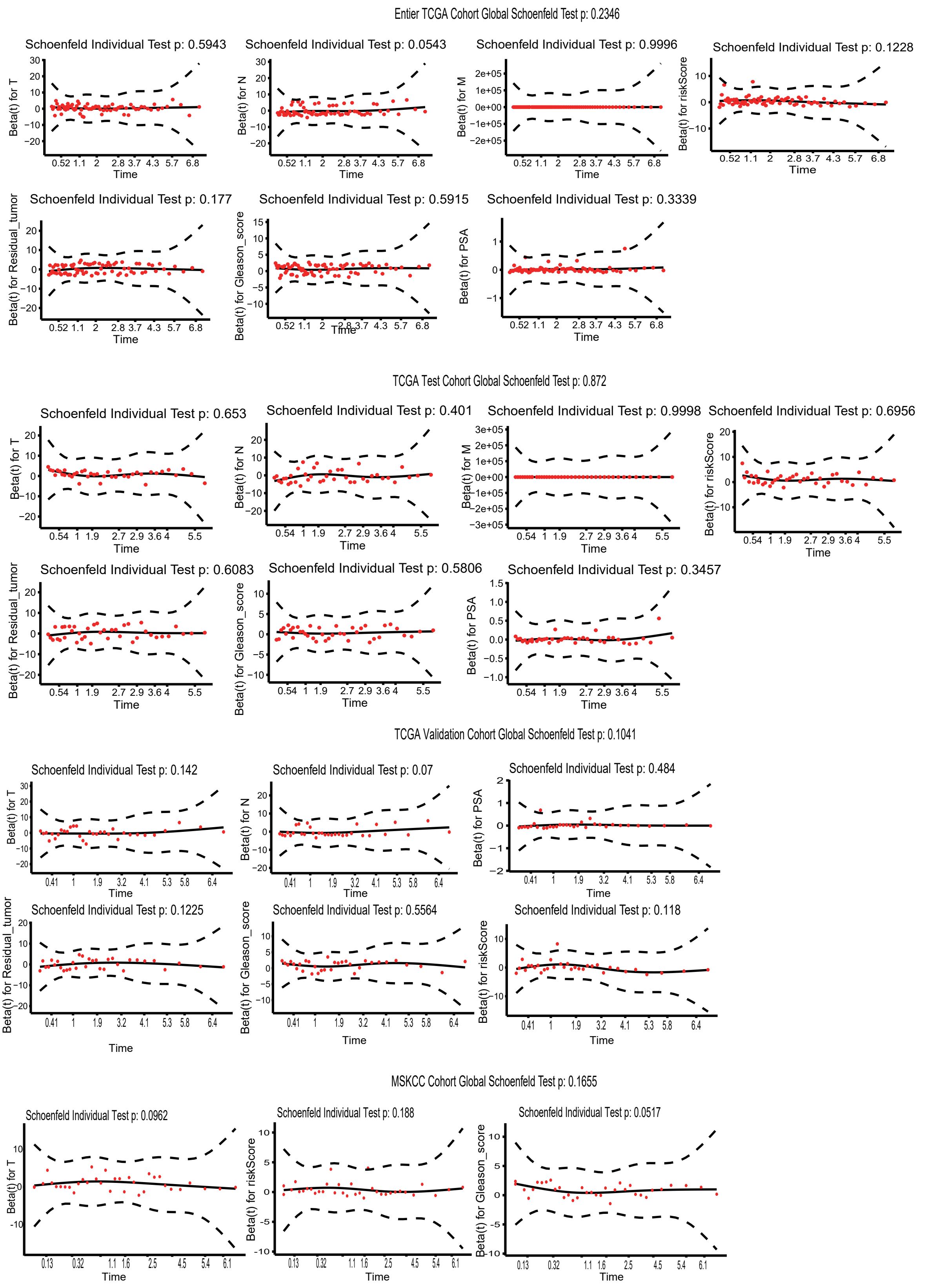

Entier TCGA Cohort Global Schoenfeld Test p: 0.2346
Schoenfeld Individual Test p: 0.5943
Beta(t) for T
Schoenfeld Individual Test p: 0.0543
Beta(t) for N
Schoenfeld Individual Test p: 0.9996
Beta(t) for M
Schoenfeld Individual Test p: 0.1228
Beta(t) for riskScore
Schoenfeld Individual Test p: 0.177
Beta(t) for Residual_tumor
Schoenfeld Individual Test p: 0.5915
Beta(t) for Gleason_score
Schoenfeld Individual Test p: 0.3339
Beta(t) for PSA
Time
TCGA Test Cohort Global Schoenfeld Test p: 0.872
Schoenfeld Individual Test p: 0.653
Beta(t) for T
Schoenfeld Individual Test p: 0.401
Beta(t) for N
Schoenfeld Individual Test p: 0.9998
Beta(t) for M
Schoenfeld Individual Test p: 0.6956
Beta(t) for riskScore
Schoenfeld Individual Test p: 0.6083
Beta(t) for Residual_tumor
Schoenfeld Individual Test p: 0.5806
Beta(t) for Gleason_score
Schoenfeld Individual Test p: 0.3457
Beta(t) for PSA
Time
TCGA Validation Cohort Global Schoenfeld Test p: 0.1041
Schoenfeld Individual Test p: 0.142
Beta(t) for T
Schoenfeld Individual Test p: 0.07
Beta(t) for N
Schoenfeld Individual Test p: 0.484
Beta(t) for PSA
Schoenfeld Individual Test p: 0.1225
Beta(t) for Residual_tumor
Schoenfeld Individual Test p: 0.5564
Beta(t) for Gleason_score
Schoenfeld Individual Test p: 0.118
Beta(t) for riskScore
Time
MSKCC Cohort Global Schoenfeld Test p: 0.1655
Schoenfeld Individual Test p: 0.0962
Beta(t) for T
Schoenfeld Individual Test p: 0.188
Beta(t) for riskScore
Schoenfeld Individual Test p: 0.0517
Beta(t) for Gleason_score
Time
